# Histamine H3 Receptor Antagonist Enhances Neurogenesis and Improves Chronic Cerebral Hypoperfusion-Induced Cognitive Impairments

**DOI:** 10.3389/fphar.2019.01583

**Published:** 2020-01-21

**Authors:** Na Wang, Jing Ma, Jing Liu, Jiangong Wang, Cuilan Liu, Hua Wang, Yong Liu, Haijing Yan, Shujun Jiang

**Affiliations:** ^1^ Department of Physiology, Binzhou Medical University, Yantai, China; ^2^ Institute for Metabolic and Neuropsychiatric Disorders, Binzhou Medical University Hospital, Binzhou, China; ^3^ Department of Pharmacy, Xinhua Hospital, Shanghai Jiaotong University School of Medicine, Shanghai, China

**Keywords:** H3R, thioperamide, chronic cerebral hypoperfusion (CCH), proliferation, cAMP-response element binding, brain-derived neurotrophic factor, neurogenesis, cognitive impairment

## Abstract

Chronic cerebral hypoperfusion (CCH) is a neurodegenerative disease, which induces cognitive impairments in the central nervous system (CNS). Histamine H3 receptor (H3R) is an autoreceptor involved in the modulation of neurogenesis and synaptic plasticity in the CNS. However, the role of H3R in CCH-induced injury and the related mechanisms remain to be clarified. Here, we found that thioperamide (THIO), a H3R antagonist, promotes the proliferation of NE-4C stem cells under either normal or oxygen-glucose deprivation (OGD) condition *in vitro*. Thioperamide promotes the phosphorylation of cAMP-response element binding (CREB), and thereby upregulates the expression and release of brain-derived neurotrophic factor (BDNF). However, H89, an inhibitor of protein kinase A (PKA)/CREB, reverses the effects of thioperamide on either BDNF expression and release or cell proliferation in NE-4C stem cells. Moreover, thioperamide has protective effects on OGD-induced impairment of cell viability and neuronal morphology in primary neurons *in vitro*. Furthermore, thioperamide enhanced neurogenesis in the dentate gyrus (DG) and subventricular zone (SVZ) regions *in vivo*, and ameliorated CCH-induced cognitive impairments. Taken together, these findings showed that thioperamide protects primary neurons against OGD-induced injury and promotes the proliferation of neural stem cells in DG and SVZ regions through CREB/BDNF pathways, thereby improving cognitive deficit.

## Introduction

Chronic cerebral hypoperfusion (CCH) is a disease in the central nervous system (CNS) that contributes to neurodegeneration and dementia (Yang et al., 2017) (Duncombe et al., 2017). CCH aggravates a variety of neurological diseases such as vascular dementia, Alzheimer’s disease (AD), and stroke (Roma, 2002), and then eventually leads to long-term memory impairment (Cechetti et al., 2012) or cognitive impairment (Gao et al., 2019). The mechanism of CCH-induced injury mainly involves the disruption of white matter integrity (Duncombe et al., 2017). However, the precise molecular and cellular mechanisms that lead to such changes are currently being unraveled, and there is no effective therapy for the treatment of CCH up to now. Therefore, it needs more efforts to clarify the mechanisms underlying CCH and explore new neuroprotective strategies.

Histamine is an endogenous neurotransmitter that regulates numerous functions in the CNS, including learning and memory, sleep/wakefulness and feeding (Witkin and Nelson, 2004; Leurs et al., 2005;). As an autoreceptor, Histamine H3 receptor (H3R) inhibits the release of histamine, and plays an important role in neuronal injuries and cognitive impairments. Inhibition of H3R can protect against ischemic injury by enhancing autophagy (Yan et al., 2014). The H3R antagonist protects against traumatic brain injury (TBI) by regulating H1R (Liao et al., 2019), and also ameliorates the cognitive deficits induced by N-methyl-D-aspartic acid receptor (NMDAR) antagonists like ketamine and MK-801 (Eissa et al., 2018). Inhibiting H3R also improves context discrimination task in aged mice (Guilloux et al., 2017). However, whether H3R antagonist is protective against CCH-induced injury and the possible mechanisms remain to be further studied.

Recently, a lot of research indicated that there are always some multipotent neural stem cells present in the adult mammalian brain, which has the potential of proliferation and differentiation throughout life (Gregoire et al., 2015). And these cells are mainly distributed in the subventricular zone of the lateral ventricles and the hippocampal subgranular zone (Sohur et al., 2012). The neuroblasts can migrate to specific areas and function accordingly (Lois et al., 1996). The adult-generated neurons play an important role in learning and memory. However, its role in pathological conditions, such as cerebral ischemia is still to be studied (Eriksson et al., 1998).

Reports show that neurogenesis that occurs in the early stage is becoming a new target for the treatment of CCH (Ahn et al., 2016). Histidine is protective against CCH-induced injury by promoting neurogenesis in the dentate gyrus (DG) region (Song et al., 2018). GW3965, a liver X receptor β agonist, ameliorates cognitive impairments induced by CCH through enhancing neurogenesis (Sun et al., 2018). The overexpression of glucagon-like peptide-2 receptor improves the spatial cognitive dysfunction induced by CCH through promoting neurogenesis (Xie et al., 2018). Exercise improves cognitive deficits through enhancing neurogenesis of CCH-induced injury (Choi et al., 2016). In this article, we have studied the effect of H3R antagonist on neurogenesis in OGD induced injury *in vitro* and CCH-induced injury *in vivo*.

## Materials and Methods

### Cell Culture

For primary cortical neuron culture, pregnant C57BL/6 mice were anesthetized with 1% pentobarbital sodium (>99%, Klontech) by intraperitoneal injection. The cortex was isolated from the embryos (18 days), and digested with trypsin (0.125%, Solarbio) for 10 min at 37°C. 800–1,000 cells/mm^2^ were seeded on plates coated with 30 mg/ml poly-d-lysine (P1399, Sigma). Cells were placed in fresh serum-free neurobasal medium (Gibco) plus 2% B27 (Gibco) and fed every 4 days with fresh medium, and then used after 7 days (DIV7). In addition, NE-4C cell line (from the ATCC), an embryonic neuroectodermal stem cell, was cultured in dulbecco’s modified eagle medium (DMEM) (Gibco) with 10% fetal bovine serum (Gibco). The cells were incubated at 37°C in a 5% CO_2_ humidified atmosphere.

### Oxygen-Glucose Deprivation (OGD)

Cells were rinsed twice with warm DMEM, and refreshed with O_2_- and glucose-free DMEM (prebalanced in an O_2_-free chamber at 37°C). Cells were then immediately placed in a sealed chamber (Billups Rothenburg, MIC-101) loaded with mixed gas containing 5% CO_2_ and 95% N_2_ for 8 min at 25 L/min. After pretreated with thioperamide for 24 h, the primary neurons were then submitted to OGD for 1 h and NE-4C cells OGD for 6 h at 37°C.

### Immunocytochemistry

Immunostaining was performed in primary cortical neurons and NE-4C cells. Cells seeded on coverslips were fixed with cold methanol (≥99.8%, yongda chemical reagent, Tianjin) for 10 min, and then incubated in 5% Bovine serum albumin (BSA, >99%, Solarbio) for 2 h to block nonspecific binding of IgG. Then the cells were reacted with primary antibody at 4°C overnight. The primary antibodies used in this experiment were rabbit monoclonal antibody against H_3_R (ab124732, Abcam, 1:200), mouse monoclonal antibody against Nestin (ab11306, Abcam, 1:200) and rabbit monoclonal antibody against MAP-2 (ab183830, Abcam, 1:100). After repeated washing in phosphate-buffered saline (PBS), cells were incubated with secondary antibody in 3% BSA for 2 h at 25°C. The secondary antibodies used in this experiment were donkey anti-rabbit IgG-Alexa Fluor 488 (A21206, Invitrogen, 1:500) and donkey anti-mouse IgG-Alexa Fluor 546 (A10036, Invitrogen, 1:500). After further washing in PBS, cultures were dried, and mounted on glass slides. The stained cells were observed under a laser scanning confocal microscope (Leica TCS SPE, Germany). Total dendritic length and neuronal complexity were quantified by using ImageJ software and the Fiji plugins Simple Neurite Tracer Analysis as well as Sholl Analysis.

### Drug Administration *In Vitro*

Thioperamide (THIO, >99%, ab120021, Abcam) is the antagonist of H3R; H89 (≥98%, B1427, Sigma-Aldrich) was used to inhibit PKA/CREB activity; and Bromodeoxyuridine (BrdU, ≥ 99%, B5002, Sigma) was used to label newly born cells.

For *in vitro* experiments, the THIO group was treated with thioperamide (1µM) for 24 h (Yan et al., 2014), and H89 (10 μM) was administrated 30 min before THIO treatment (Song et al., 2015). The examination of the effect of THIO on p-CREB and BDNF expression under OGD-induced injury was carried out at the end of the OGD treatment. After administrating with thioperamide for 18 h, BrdU (10 μM) was added to the media during the last 6 h (Garza et al., 2012).

### MTT Assay

The viability of cells was measured by 3-(4,5-dimethylthiazol-2-yl)-2,5- diphenyltetrazolium bromide (MTT, Sigma) assay. Brieﬂy, cells were incubated with 0.5 mg/ml MTT for 2 h, the supernatant layer was then removed, and 100 µl/well of dimethyl sulfoxide (DMSO, Solarbio) was added into the 96-well plates. MTT metabolism was quantitated spectrophotometrically at 570 nm in a Biorad microplate reader. Results were expressed as the percentage of MTT reduction, taking the absorbance of control cells as 100%.

### Cell Proliferation Assay by BrdU Staining

For *in vitro* experiments, the NE-4C cells were digested and seeded on coverslips, then fixed with cold methanol for 10 min. After incubating in 2 N HCl for 30 min at 37°C and neutralized with 0.1 M borate buffer (Sinopharm Chemical Reagent, PH = 8.5) for 10 min, cells were incubated in 1% H_2_O_2_ (30% H1009, Sigma) for 10 min and then blocked with PBS containing 1% BSA and 0.3% (*w/v*) Triton X-100 (T8787, Sigma) for 1 h at room temperature. They were then reacted with rat monoclonal anti-BrdU (ab6326, Abcam, 1:50) overnight at 4°C. After washing with PBS, they were incubated with donkey anti-rat Alexa Fluor 594 (A21208, Invitrogen, 1:1,000) for 2 h at room temperature, then reacted with DAPI (C1006, Beyotime) for 5 min.

For *in vivo* experiments, the frozen sections (20 μm) were returned to room temperature, and washed 3 times with PBS. In addition to incubating DAPI, the following steps were the same as above. The stained cells were observed under a laser scanning confocal microscope (Leica TCS SPE, Germany). The cell proliferation rate was indicated as BrdU^+^ cells/total cells × 100%.

### Examination of BDNF Levels by ELISA Assay

After drug administration, the cellular supernatant of NE-4C cells was collected and centrifuged at 1,000×g at 4°C for 10 min. The supernatant was collected and the concentration of BDNF was examined by using ELISA kit (SEKM-0143, Solarbio) according to the product specification.

### Western Blot

The NE-4C cells were lysed in ice-cold RIPA lysis buﬀer (R0020, Solarbio), then centrifuged at 14,000×g at 4°C for 20 min, and the protein concentration in the extracts was determined by the Bradford assay (Thermo, Hercules, CA). The precipitates were denatured with SDS sample loading buﬀer and separated on 10% SDS PAGE. Proteins were transferred onto nitrocellulose membranes using a Bio-Rad mini-protein-III wet transfer unit for 90V/90 min. Transfer membranes were then incubated with blocking solution (5% nonfat dried milk dissolved in tris buﬀered saline tween (TBST) buﬀer (in mM): 10 Tris (≥99.8%, Sinopharm Chemical Reagent)-HCl (36-38%, Yantai sanhe chemical reagent), 150 NaCl (≥99.5%, Sinopharm Chemical Reagent), and 0.1% Tween-20 (≥40%, Sigma) for 2 h at room temperature, and incubated with primary antibody overnight at 4°C. The primary antibodies used in this experiment were phospho-CREB (9198S, Cell Signaling Technology, 1:1,000), BDNF (ab108319, Abcam, 1:1,000) and GAPDH (KC-5G4, KangChen Bio-tech, 1:1,000). Membranes were washed three times in TBST buﬀer and incubated with the appropriate secondary antibodies (Odyssey, LI-COR, 1:5,000 dilution) for 2 h. Images were acquired with the Odyssey infrared imaging system and analyzed as specified in the Odyssey software manual. The results were expressed as the target protein/GAPDH ratio and then normalized to the values measured in the control groups (presented as 100%).

### Animals

Adult male C57BL/6 mice (Pengyue Laboratory, Jinan, China) weighing 22–25 g were used in this study. The mice were housed in a temperature- and humidity-controlled animal facility, which was maintained on a 12-h light/dark cycle, food and water were given *ad libitum*. All animal studies were carried out according to protocols approved by the Institutional Animal Care and Use Committee of Binzhou Medical University, and conducted in compliance with the National Institutes of Health Guide for the Care and Use of Laboratory Animals. The approval number for the animal experiments is SYXK (Lu) 2018 0022. Eﬀorts were made to minimize any pain or discomfort, and the minimum number of animals was used.

### Chronic Cerebral Hypoperfusion

Mice in CCH were implemented by interdicted blood flow to the right common carotid artery as described previously (Yoshizaki et al., 2008). Briefly, mice were anesthetized by intraperitoneal injection of 1% pentobarbital sodium, and maintained the body temperature at 37°C during surgery with the heat lamp. Separated the right common carotid artery from the contiguous vagus nerve, then ligated the right CCA with two 6-0 silk sutures, and cut the right CCA between the two knots. For sham mice, the steps were the same as those in the experimental group except for the ligation and cut of the right CCA.

### Drug Administration *In Vivo*

For *in vivo* experiments, after CCH surgery, thioperamide (i.p., 5 mg/kg) was administrated twice (at hypoperfusion and 6 h later) on the first day and then treated every two days until the behavior experiments begun on day 25 (Yan et al., 2014). BrdU (i.p., 50mg/kg) was injected immediately after CCH surgery for 4 times every 4 h (Gruneberg et al., 2016), and the mice were sacrificed at either 24 h after the last injection or 35 days after surgery.

### BrdU/NeuN Staining

BrdU was used to label newly born cells and NeuN was used to label mature neurons. The frozen brain sections (20 μm) were recovered to the room temperature, and washed with PBS. Then they were incubated for 30 min in 2 N HCl at 37°C, and neutralized with 0.1 M borate buffer (Sinopharm Chemical Reagent, PH = 8.5) for 10 min. After incubating in 1% H_2_O_2_ (30% H1009, Sigma) for 10 min, the sections were blocked with PBS containing 1% BSA and 0.3% (*w/v*) Triton X-100 (T8787, Sigma) for 1 h at room temperature. They were then reacted with rat monoclonal anti-BrdU (ab6326, Abcam, 1:50) and rabbit monoclonal anti-NeuN (24307T, CST, 1:50) at 4°C overnight. After washing with PBS, they were incubated with donkey anti-rat Alexa Fluor 594 (A21208, Invitrogen, 1:1000) and donkey anti-rabbit IgG-Alexa Fluor 488 (A21206, Invitrogen, 1:500) for 2 h at room temperature. The stained cells were observed under a laser scanning confocal microscope (Leica TCS SPE, Germany). Image analysis was performed using Image J software.

### Stereological Cell Counting of BrdU^+^ Cells

We used the stereological techniques to count the number of BrdU^+^ cell as described previously (Wang et al., 2015). BrdU^+^ cells from every eighth section covering the entire rostrocaudal axis of the DG were counted using a high-power (40×) microscope. Cells were counted in a blind manner. At least eight sections from both sides of the DG were counted per animal. The number for each group of animals is indicated in figure legends.

### Novel Object Recognition

The novel object recognition (NOR) test was performed on day 25–28 after surgery. The NOR test was performed as previously described (Zhang et al., 2018). Briefly, mice were placed in a 40*40*30 cm arena to be familiar with the environment for 8 min every day on day 25 and day 26. Then, two objects of different colors and shapes were placed in the arena on day 27, and replaced one of the objects with a brand new one on day 28. The mice were placed in the arena for 10 min and the exploring times on the two objects were recorded respectively on day 28. The discrimination index (DI) was calculated as: DI (%) = (time spent with novel object - time spent with old object)/(time spent with novel object + time spent with old object) × 100%.

### Y Maze

The Y maze (YM) test was performed as previously described (Chiba et al., 2009) on day 29 after surgery. Briefly, the apparatus for YM was made of gray plastic, with each arm 40 cm long, 12 cm high, 3 cm wide at the bottom, and 10 cm wide at the top. The three arms were connected at an angle of 120°. Mice were individually placed at the end of an arm and allowed to explore the maze freely for 8 min. The total arm entries and spontaneous alternation percentage (SA%) were measured. SA% was defined as a ratio of the arm choices that differed from the previous two choices (“successful choices”) to total choices during the run (“total entry minus two” because the first two entries could not be evaluated). For example, if a mouse made 10 entries, such as 1-2-3-2-3-1-2-3-2-1, there are five successful choices in eight total choices (10 entries minus 2). Therefore, SA% in this case is 62.5%.

### Morris Water Maze

The Morris water maze (MWM) maze test was performed as previously described on day 30–35 after surgery (Vorhees and Williams, 2006). Briefly, the water maze of 1.50 m in diameter and 0.50 m in height was filled with water (20 ± 1°C) to maintain the water surface 1.50 cm higher than the platform (10 cm in diameter). Water was dyed white and the tank was divided into four quadrants by four points: North (N), South (S), East (E), and West (W). The platform was placed at the center of either quadrant and video tracking software was used to automatically track the animals. Learning and memory acquisition was started on day 30 and lasts for five days. Animals were put into the water from four points in random order every day until they found the platform and stayed for 10 s within 1 min. If the mice cannot find the platform within 1 min, they were guided to the platform. Learning and memory maintenance phase begun on day 35. The platform was removed, and the mice were placed in water from the opposite quadrant of the platform, and then the times crossing the platform was recorded within 1 min.

### Statistical Analyses

Results are expressed as mean ± SEM. Statistical analysis was performed by one-way ANOVAs followed by Tukey’s *post hoc* comparisons or two-way ANOVAs followed by Bonferroni *post hoc* comparisons, using prism software. *P* value < 0.05 was considered statistically significant.

## Results

### Effect of Thioperamide on Proliferation of NE-4C Cells

**Figure1:**
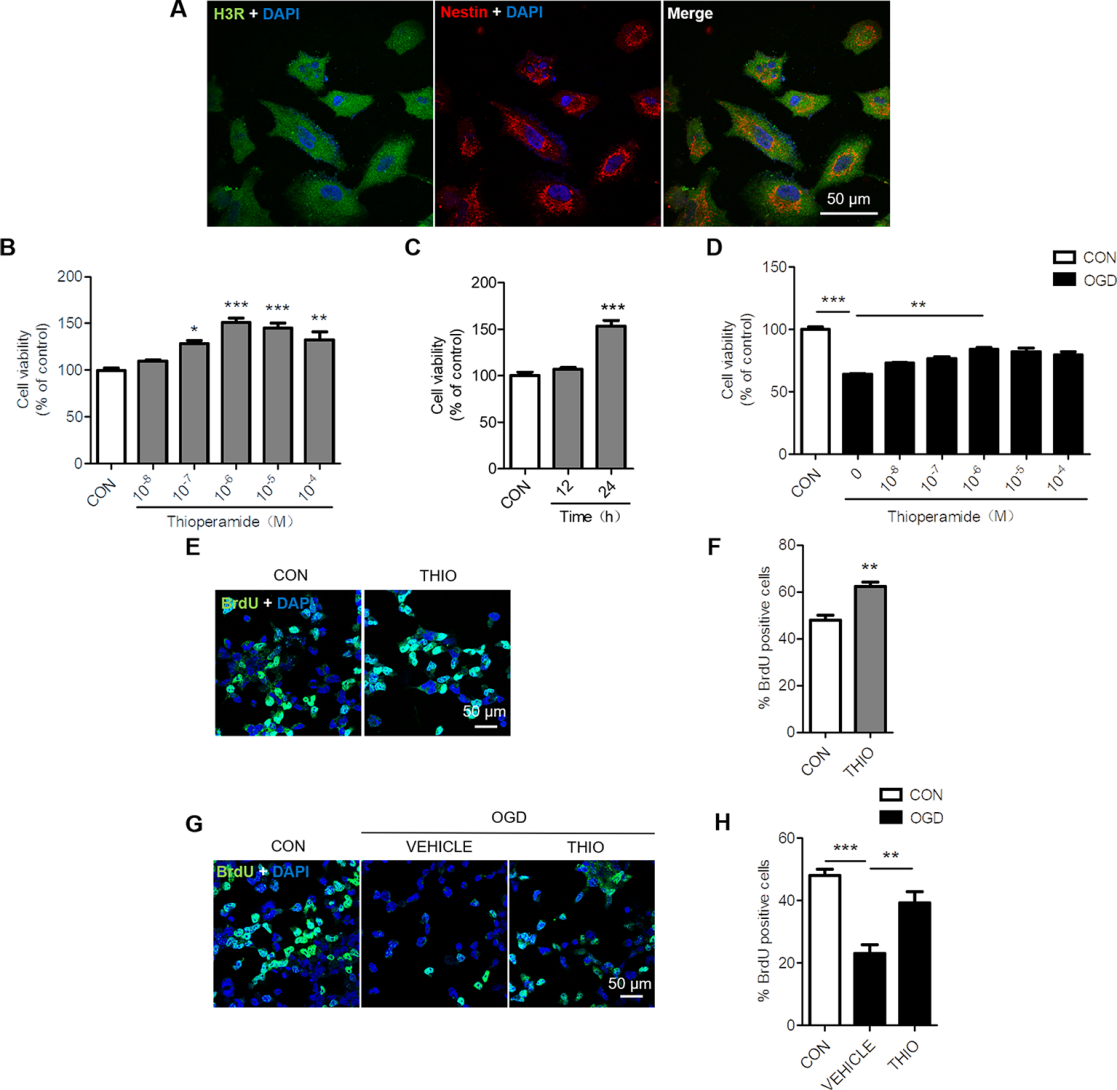
Effect of thioperamide on the proliferation of NE-4C stem cells. **(A)** Representative immunocytochemical staining showing colocalization of H3R (green) and nestin (red) in NE-4C stem cells. Scale bar, 50 μm. **(B, C)** Cell viability by (MTT assay showing the effect of thioperamide (THIO) with different concentrations **(B)** and different incubation times **(C)** on viability of NE-4C stem cells under normal condition. **(D)** Cell viability showing different concentrations of thioperamide with incubation of 24 h under OGD-induced injury in NE-4C stem cells. **(E, F)** Representative immunocytochemical staining of BrdU (green) along with DAPI (blue) **(E)** and bar graph **(F)** showing the effect of thioperamide on proliferation of NE-4C stem cells under normal condition. Scale bar, 50 μm. **(G, H)** Representative immunocytochemical staining of BrdU (green) along with DAPI (blue) **(G)** and bar graph **(H)** showing the effect of thioperamide on proliferation of NE-4C stem cells under OGD-induced injury. Scale bar, 50 μm. Data are presented as mean ± SEM. n = 18 for each group in **(B–D)**. Results normalized relative to the control group (regard as 100%). n = 6 for each group in F and H. *P < 0.05; **P < 0.01; ***P < 0.001.

To determine whether H3R is expressed in NE-4C cells, we examined the expression of H3R by using Immunocytochemical staining. The results indicated that H3R was found in nestin positive cells, a marker for neural progenitor cells (**Figure 1A**). Reports have shown that H3R antagonist promotes hippocampal neurogenesis in aged mice (Guilloux et al., 2017). To investigate the role of H3R on neural stem cells, NE-4C cells were used. The MTT results indicated that thioperamide, a H3R antagonist, promoted the viability of NE-4C stem cells in a concentration-dependent manner. The viability of NE-4C stem cells increased significantly to 150.83 ± 6.91% (*P* < 0.001, **Figure 1B**) when thioperamide (10^-6^ M) was administrated, and increased to 145.11 ± 14.52% (*P* < 0.001, **Figure 1B**) and 132.02% ± 25.65% (*P* < 0.01, **Figure 1B**) when 10^-5^ M and 10^-4^ M of thioperamide were administrated respectively.

Moreover, the time course of the effects of thioperamide on viability of NE-4C cells was determined after treatment with thioperamide (10^-6^ M). The results suggested that thioperamide promoted cell viability significantly to 153.24% ± 22.10% (*P <* 0.001, **Figure 1C**) when incubated for 24 h, however, there was no significant difference in cell viability when incubated for 12 h (*P >* 0.05, **Figure 1C**). Therefore, a dose of 10^-6^ M and the incubated time of 24 h were selected for the later study.

H3R was reported to aggravate cerebral ischemic brain injury (Yan et al., 2014). In order to investigate the role of H3R on neural stem cells under ischemic injury, an OGD-induced injury *in vitro* was used. The MTT results showed that the cell viability declined significantly to 63.90% ± 3.03% (*P* < 0.001, **Figure 1D**) under OGD for 6 h, and thioperamide rescued NE-4C from OGD-induced cell injury, as cell viability increased significantly to 84.36% ± 3.96% (*P* < 0.01, **Figure 1D**) when thioperamide (10^-6^ M) was administrated for 24 h before OGD.

In order to further investigate the cell proliferation offered by thioperamide under normal circumstances and OGD-induced injury, BrdU staining on NE-4C stem cells was used (Keilhoff et al., 2014). Results showed that thioperamide remarkably increased the rate of BrdU^+^ cells from 48.04% ± 4.23% to 62.30% ± 3.74% under normal circumstances (*P <* 0.01, **Figures 1E, F**). Moreover, the rate of BrdU^+^ cells declined significantly to 23.16% ± 5.29% (*P* < 0.001, **Figures 1G, H**) under OGD-induced injury, which was rescued by thioperamide to 39.23% ± 7.16% (*P* < 0.01, **Figures 1G, H**). Above all, these results suggested that thioperamide promoted the viability and proliferation of neural stem cells under both normal circumstances and OGD-induced injury.

### Involvement of CREB/BDNF Pathway in the Effect of Thioperamide on Proliferation in NE-4C Cells

**Figure 2:**
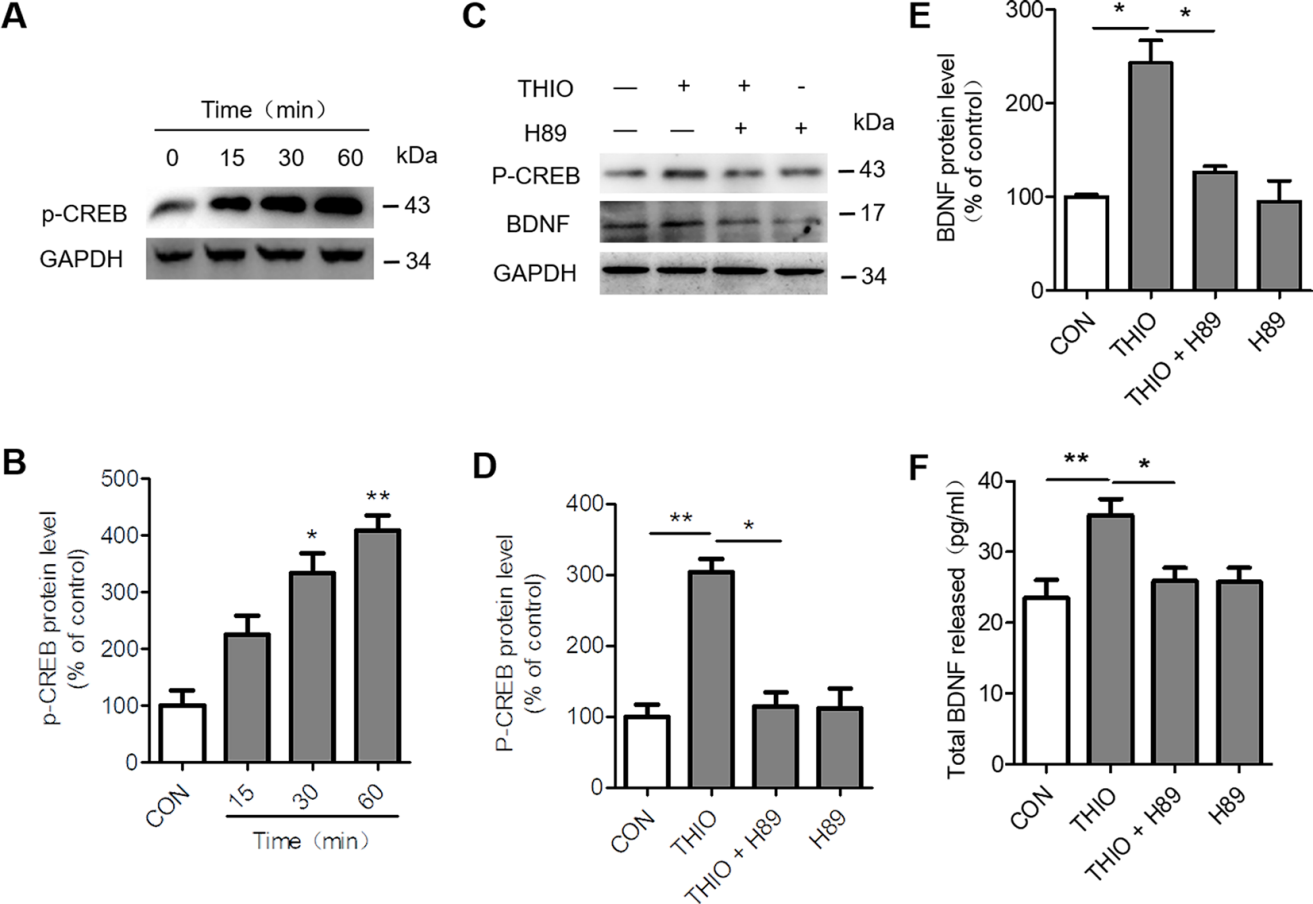
Effect of thioperamide on the cAMP-response element binding (CREB)/brain-derived neurotrophic factor (BDNF) pathway in NE-4C stem cells. **(A**, **B)** Representative western blot image **(A)** and quantitative bar graph **(B)** showing the effect of thioperamide (THIO) with different incubation times on the expression of p-CREB **(B)**. **(C–E)** Representative western blot image **(C)** and quantitative bar graph **(D, E)** showing the reversed effect of H89 on the expression of p-CREB **(D)** and BDNF by thioperamide (incubated for 24 h) **(E)**. **(F)** The effect of H89 and thioperamide (incubated for 24 h) on released BDNF levels was tested by ELISA. Data are presented as mean ± SEM. Results normalized relative to the control group (regard as 100%). n = 7 for each group in **(B, C)**; n = 6 for each group in **(D, F, G** and **H)**. *P < 0.05, **P < 0.01.

H3R is a G-protein-coupled receptor that activates Gi/o proteins to inhibit adenylyl cyclase (AC) activity and the formation of cyclic AMP (cAMP). cAMP activates protein kinase A (PKA) and subsequently cAMP-responsive-element-binding protein (CREB) to modulate gene transcription. As a result, H3R activation lowers cAMP levels and reduces CREB-dependent gene transcription (Leurs et al., 2005). Moreover, CREB phosphorylation promotes neurogenesis through upregulating BDNF (Yang et al., 2015). In order to investigate the effect of thioperamide on CREB/BDNF pathway, we analyzed the p-CREB and BDNF expression when thioperamide was administrated in NE-4C cells (**Figure 2A**). The Western blots results showed that the expression of p-CREB increased to 333.39% ± 49.52% (*P* < 0.05, **Figure 2B**) when thioperamide was incubated for 30 min, and significantly increased to 408.72% ± 38.87% (*P* < 0.01, **Figure 2B**) when incubated for 60 min.

To further investigate the involvement of CREB/BDNF pathway in the promoted proliferation of thioperamide on NE-4C cells under normal circumstances and OGD-induced injury, H89, a PKA/CREB inhibitor was used (Umeda et al., 2008). Firstly, we assessed the effect of H89 on the expression of p-CREB and BDNF. The results showed that H89 significantly reversed the upregulated level of p-CREB (from 304.00% ± 26.64% to 109.91% ± 34.79%, *P <* 0.05, **Figures 2C, D**) and BDNF (from 202.87% ± 33.95% to 126.11% ± 9.09%, *P <* 0.05, **Figures 2C, E**) by thioperamide. Moreover, H89 also reversed the increased concentration of BDNF in the thioperamide group from 35.22 ± 6.37 pg/ml to 25.80% ± 5.61% pg/ml (*P <* 0.05, **Figure 2F**).

**Figure 3:**
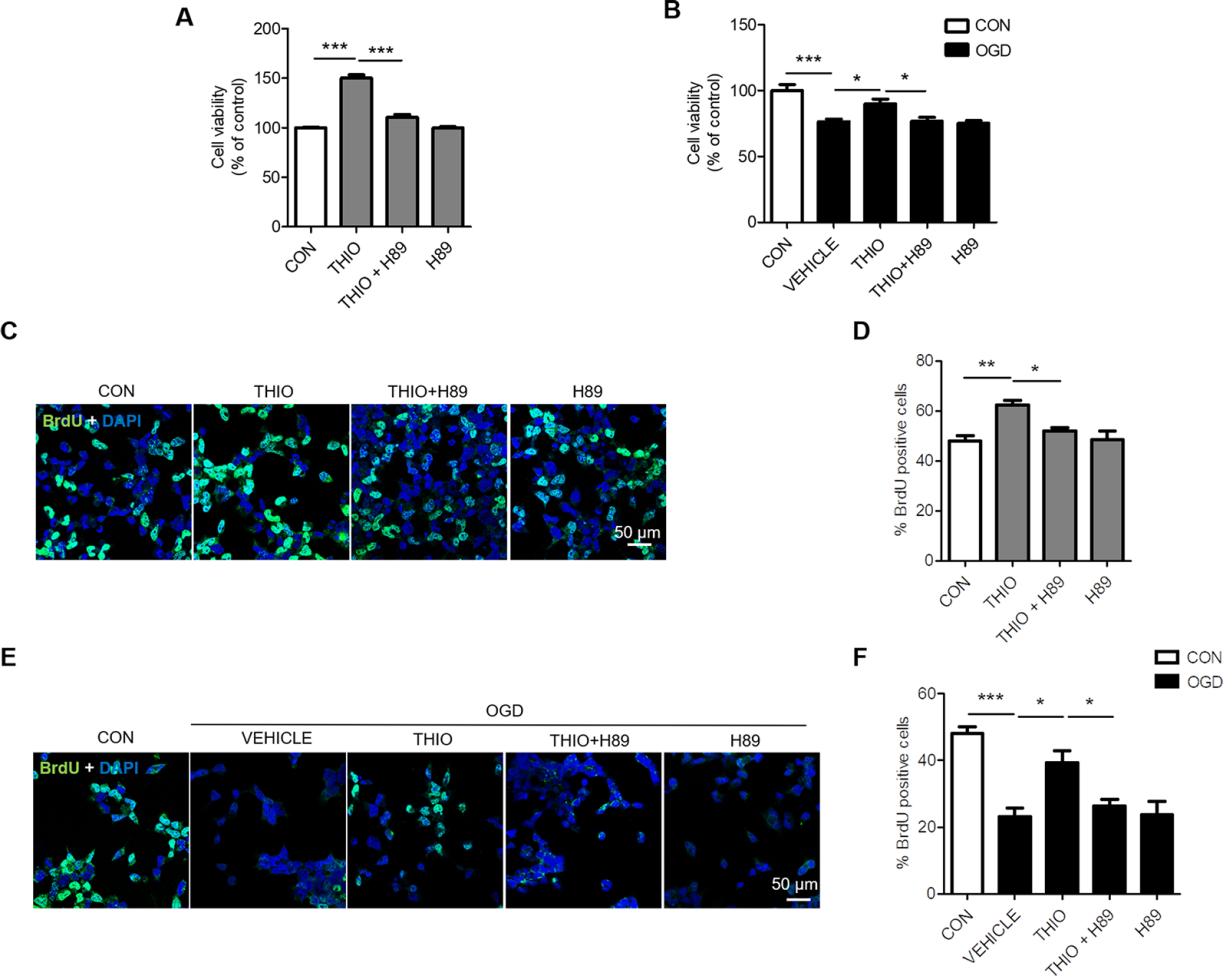
Inhibition of cAMP-response element binding (CREB)/brain-derived neurotrophic factor (BDNF) by H89 reversed the promoted proliferation by thioperamide in NE-4C stem cells. **(A, B)** Cell proliferation by MTT assay showing the effect of thioperamide (THIO) and H89 on proliferation of NE-4C stem cells under either normal **(A)** or OGD **(B)** circumstances. **(C—F)** Representative immunocytochemical staining of BrdU (green) along with DAPI (blue) **(C, E)** and bar graph **(D, F)** showing the effect of thioperamide on proliferation of NE-4C stem cells under under either normal **(C, D)** or OGD **(E, F)** circumstances. Scale bar, 50 μm. Data are presented as mean ± SEM. n = 12 for each group in **A** and **B**. Results normalized relative to the control group (regard as 100%). n = 6 for each group in **(D, F)**. *P < 0.05, **P < 0.01, ***P < 0.001.

Furthermore, we examined the effect of H89 on the promoted proliferation on neural stem cells. The results indicated that cell proliferation decreased significantly in the thioperamide + H89 group compared with the thioperamide group (from 150.33% ± 10.60% to 110.35% ± 10.89%, *P <* 0.05, **Figure 3A**) under normal circumstance. Thioperamide also markedly rescued proliferation of NE-4C stem cells from OGD-induced injury (from 58.88% ± 8.43% to 89.96% ± 13.45%, *P <* 0.001, **Figure 3B**), which was reversed significantly by H89 (to 75.88% ± 9.35%, *P <* 0.05, **Figure 3B**). In addition, BrdU staining was also carried on to assess the effect of thioperamide and H89 on neural stem cells proliferation. Results showed that the increased rate of BrdU^+^ cells offered by thioperamide was reserved significantly by administration of H89 (from 62.30% ± 3.74% to 52.05% ± 2.61%, *P <* 0.05, **Figures 3C, D**) under normal circumstance. Under the OGD-induced injury, the rate of BrdU^+^ cells decreased from 48.04% ± 4.23% to 23.16% ± 5.29% (*P <* 0.001, **Figures 3E, F**), and thioperamide promoted the rate of BrdU^+^ cells to 39.23% ± 7.16%, (*P <* 0.05, **Figures 3E, F**), which was reversed by administration of H89 to 26.30% ± 4.16% (*P <* 0.05, **Figures 3E, F**). In addition, H89 have no significant effect on proliferation under OGD-induced injury. Results above revealed that thioperamide might promote the proliferation of NE-4C stem cells at least partly through CREB/BDNF pathway.

### Effect of Thioperamide on Neuronal Viability and Morphology Under OGD-Induced Injury in Primary Neurons

**Figure 4:**
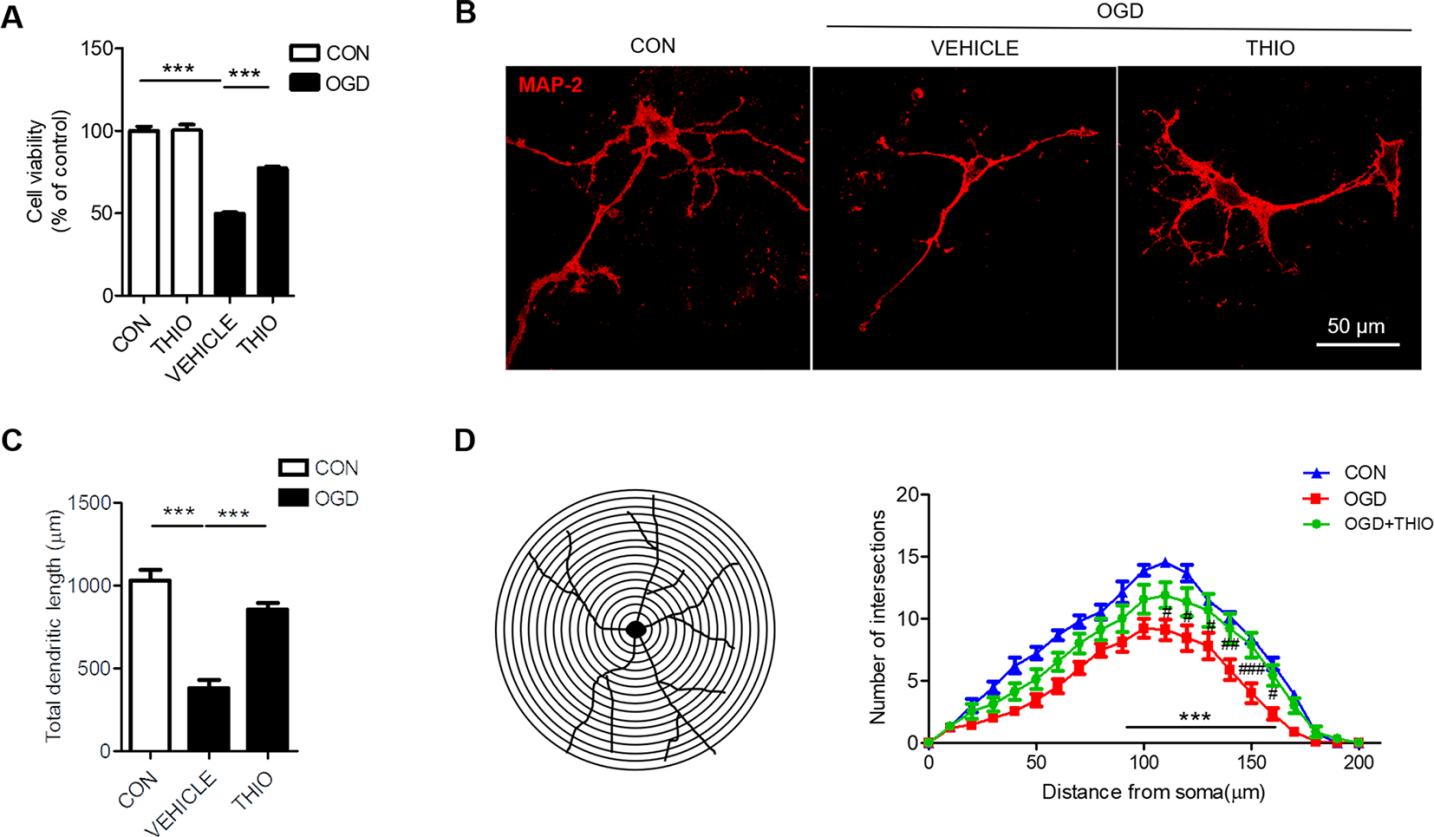
Effect of thioperamide on OGD-induced impaired cell viability and dendritic morphology in primary neurons**. (A)** Effect of thioperamide (THIO) on cell viability under normal and OGD condition in primary neurons. **(B–D)** Representative immunocytochemical staining of MAP-2 (red) **(B)** and quantitative bar graph **(C, D)** showing the effect of thioperamide on neuronal morphology including total dendritic length **(C)** and dendritic intersections **(D)** under OGD-induced injury. Scale bar, 50 μm. Data are presented as mean ± SEM. n = 12 for each group in **(A)** n = 9 for each group in **(C, D)**. ***P < 0.001 in **(A, C)**. ***P < 0.001 vs. the control group; ^#^ *P < *0.05, ^##^ *P* < 0.01, ^###^ *P < *0.001 vs. the OGD group in **(D)**.

To investigate the effect of thioperamide on neurons under OGD, we assessed the cell viability in primary neurons. The results indicated that the thioperamide had no effect on the viability of neurons under normal condition (*P* > 0.05, **Figure 4A**), and markedly reduced the viability to 49.92% ± 2.00% (*P < *0.001, **Figure 4A**) under OGD for 1 h, and thioperamide (10^-6^ M) significantly protected the OGD-induced injury (from 49.92% ± 2.00% to 77.37% ± 3.45%, *P <* 0.001, **Figure 4A**).

It is reported that ischemic injury induces morphological changes of dendrite (Trinchero et al., 2017; Liu et al., 2019). Thus, we investigated the role of thioperamide on neuronal morphology by MAP-2 staining. The results showed that the total dendritic length was 383.67 μm ± 146.15 μm in the OGD group compared with 1034.89 μm ± 182.39 μm in the control group (*P <* 0.001, **Figures 4B, C**). Thioperamide rescued the total dendritic length to 855.22 μm ± 3.45 μm (*P <* 0.001, **Figures 4B, C**). We further investigated the effect of thioperamide on dendritic complexity. The Sholl analysis indicated that the dendritic intersections reduced significantly between 110 μm and 160 μm from the soma in the OGD group compared with the control group (*P <* 0.01, **Figures 4B, D**). Thioperamide significantly increased the dendritic intersections between 90 μm and 160 μm from the soma compared with the OGD group (*P <* 0.001, **Figures 4B, D**). Therefore, the above results revealed that thioperamide protected the primary neurons against OGD-induced injury, and increased the total dendritic length and dendritic complexity on OGD-induced injury.

### Effect of Thioperamide on Proliferation of Neural Stem Cells on the Early Stage Under CCH-Induced Injury *In Vivo*

**Figure 5:**
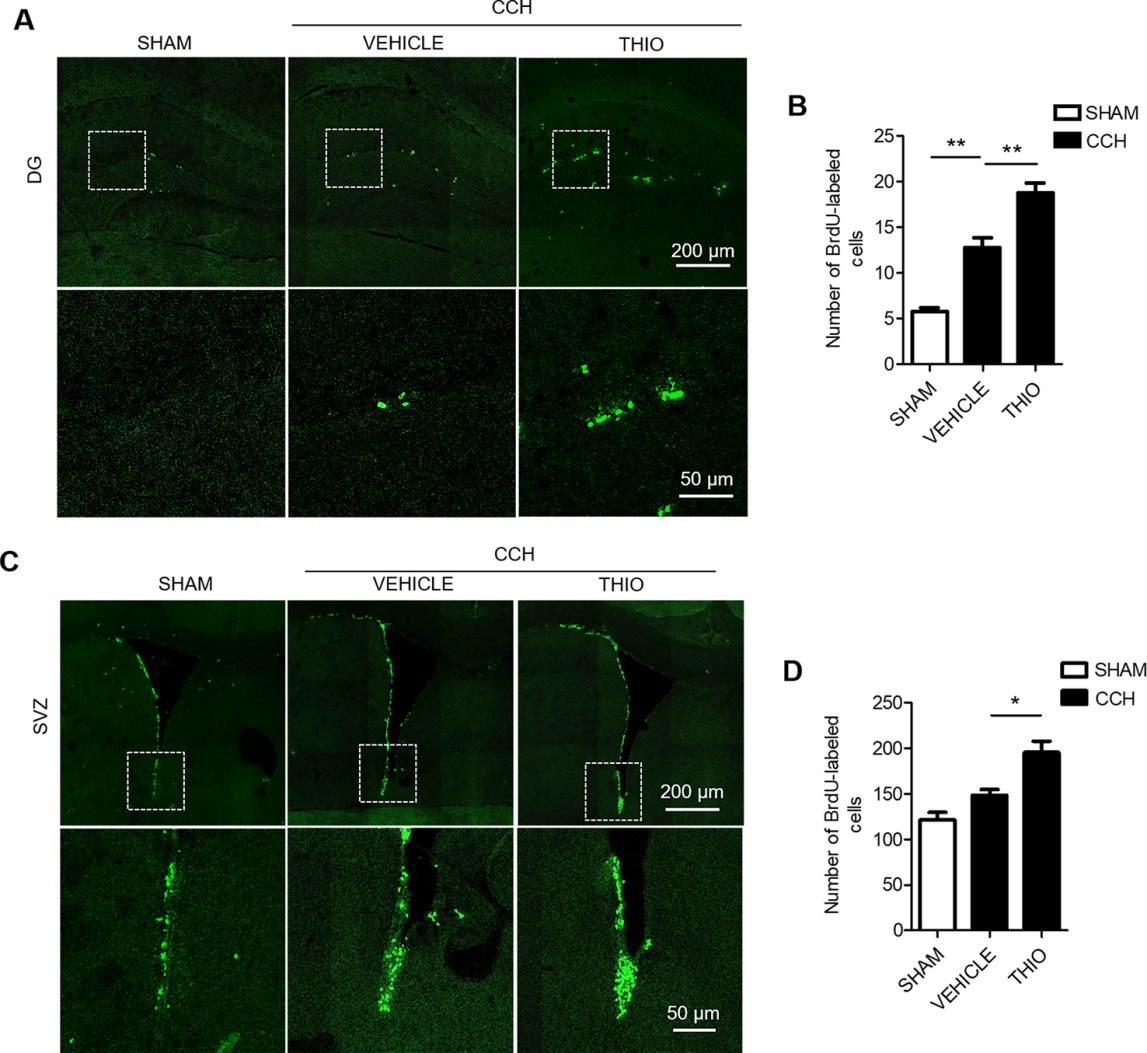
Effect of thioperamide on chronic cerebral hypoperfusion (CCH)-induced proliferation of NSC cells in dentate gyrus (DG) and subventricular zone (SVZ) regions on day 2. Representative immunohistochemical staining of BrdU^+^ (green) **(A, C)** and quantitative bar graph including BrdU^+^ cells **(B, D)** showing the effect of thioperamide (THIO) on newborn cells under CCH-induced injury in both DG **(A, B)** and SVZ **(C, D)** regions. Scale bar: up, 200 μm; down, 50 μm. Data are presented as mean ± SEM. n = 12 for each group. *P < 0.05, **P < 0.01.

In order to study the effect of thioperamide on the proliferation of CNS cells on the early stage of chronic ischemic injury, CCH model was used. The results indicated that the BrdU^+^ cells in the DG region increased significantly in the vehicle group compared to the sham group (from 5.75% ± 16.69% to 12.75% ± 17.41%, *P <* 0.01, **Figures 5A, B**), and thioperamide markedly increased the BrdU^+^ cells to 18.75% ± 11.84% compared with the vehicle group (*P <* 0.01, **Figures 5A, B**). In the SVZ region, there was no significantly difference of BrdU^+^ cells between the vehicle group and the sham group (*P >* 0.05, **Figures 5C, D**), but thioperamide significantly increased the BrdU^+^ cells compared with the vehicle group (from 148.50% ± 8.20% to 195.5% ± 12.69%, *P <* 0.01, **Figures 5C, D**). The results above suggested that CCH promoted the proliferation of CNS cells, and thioperamide further promoted the proliferation in the DG and the SVZ region. The promoted effect of thioperamide on proliferation on day 2 after CCH surgery might be an endogenous injury protection mechanism.

### Effect of Thioperamide on Neurogenesis on the Later Stage Under CCH-Induced Injury *In Vivo*

**Figure 6:**
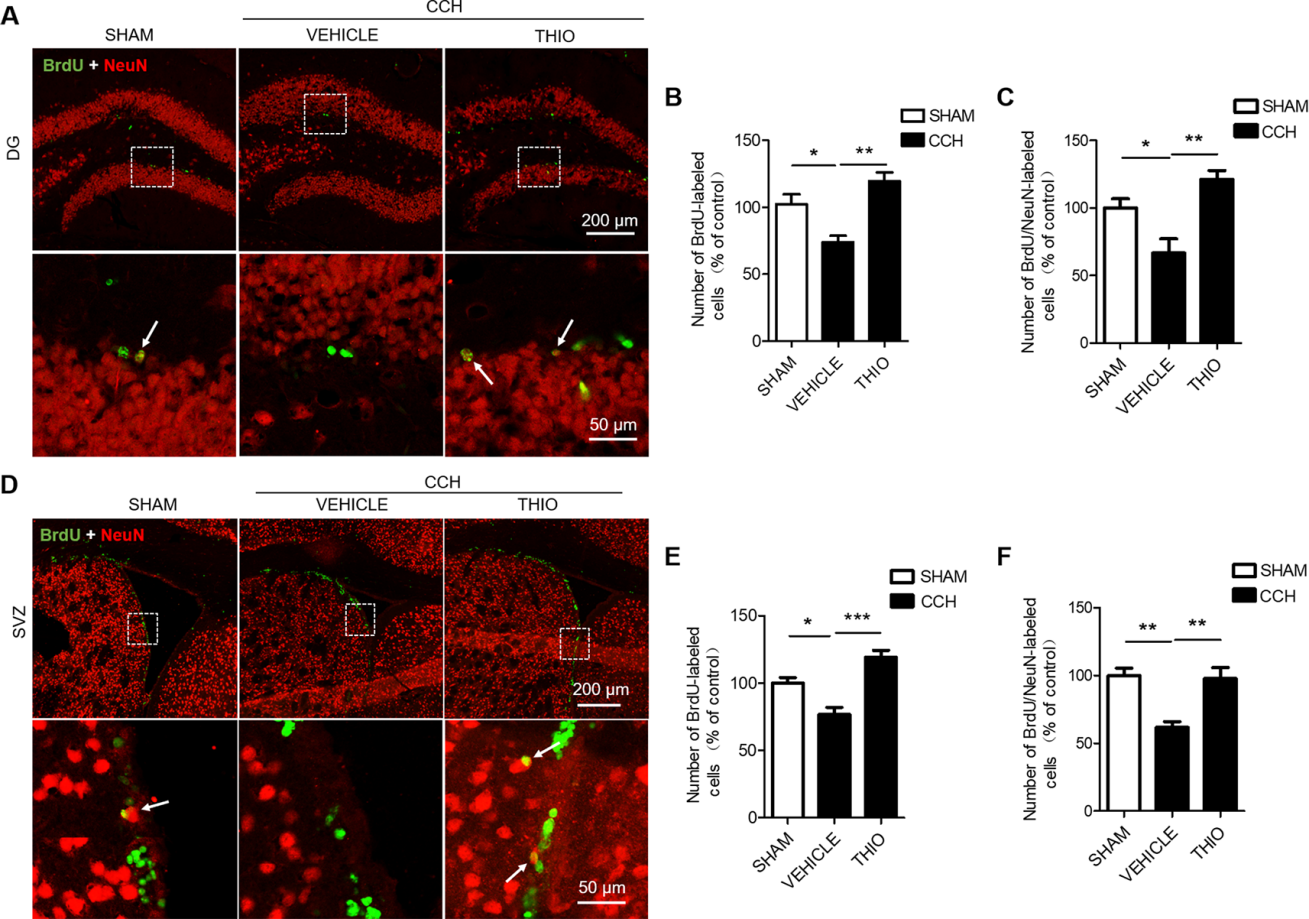
Effect of thioperamide on chronic cerebral hypoperfusion (CCH)-induced impaired neurogenesis in dentate gyrus (DG) and subventricular zone (SVZ) regions *in vivo*. Representative immunohistochemical staining of BrdU^+^ (green) and NeuN (red) **(A, D)** and quantitative bar graph including BrdU^+^ cells **(B, E)** and rate of BrdU^+/^NeuN^+^ **(C, F)** showing the effect of thioperamide (THIO) on newborn cells under CCH-induced injury in both DG **(A**–**C)** and SVZ **(D**–**F)** regions. The white arrow indicates a double labeling. Scale bar: up, 200 μm; down, 50 μm. Data are presented as mean ± SEM. n = 12 for each group. *P < 0.05, **P < 0.01, ***P < 0.001.

To investigate the effect of thioperamide on neurogenesis under chronic ischemic injury, mice were sacrificed on day 35 and BrdU^+/^NeuN^+^ staining was used. The results indicated that the BrdU^+^ cells in the DG region decreased significantly in the vehicle group to 73.80% ± 9.67% of the sham group (*P* < 0.05, **Figures 6A, B**), and thioperamide markedly increased the BrdU^+^ cells to 119.57% ± 13.12% (*P* < 0.01, **Figures 6A, B**). In the SVZ region, the CCH-induced injury also induced a decreased number of the BrdU^+^ cells to 76.65% ± 10.57% of the sham group (*P <* 0.05, **Figures 6D, E**), and thioperamide significantly increased the BrdU^+^ cells to 119.15% ± 10.35% (*P <* 0.001, **Figures 6D, E**). Moreover, the neurogenesis was shown by BrdU^+/^NeuN^+^ staining. Results suggested that BrdU^+/^NeuN^+^ cells in the DG region decreased significantly in the vehicle group to 66.67% ± 21.05% of the sham group (*P* < 0.05, **Figures 6A, C**), which was rescued by thioperamide to 121.05% ± 13.59% (*P <* 0.01, **Figures 6A, C**). In the SVZ region, similarly, CCH-induced impaired neurogenesis, as it was revealed that the BrdU^+/^NeuN^+^ cells declined to 61.89% ± 8.27% of the sham group (*P <* 0.01, **Figures 6D, F**), which was significantly reversed by thioperamide to 97.78% ± 16.23% (*P* < 0.01, **Figures 6D, F**). These results suggested that thioperamide promoted neurogenesis in the DG and the SVZ region under CCH-induced injury.

### Thioperamide Alleviated the CCH-Induced Cognitive Impairments

**Figure 7:**
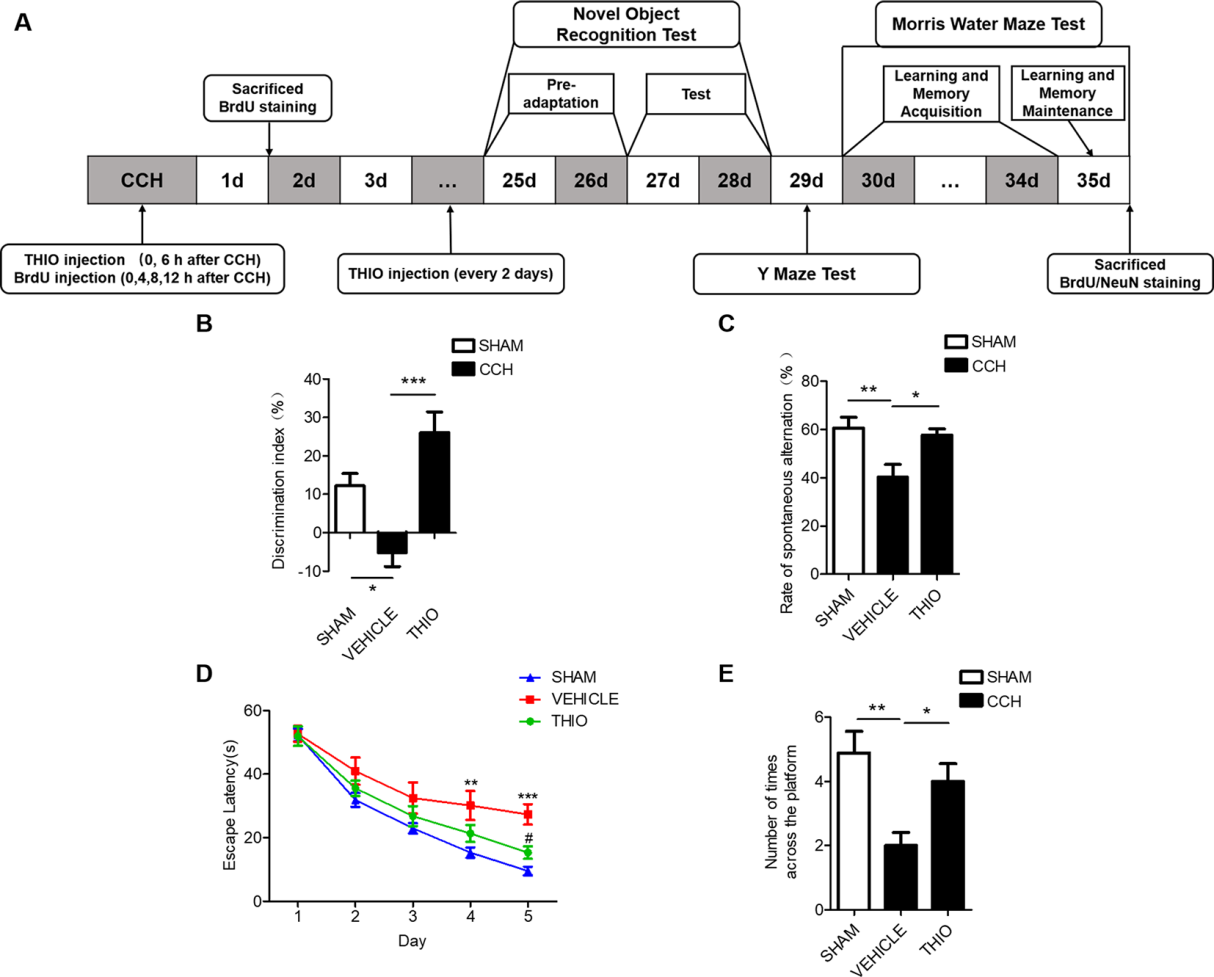
Effect of thioperamide on chronic cerebral hypoperfusion (CCH)-induced cognitive impairments. **(A)** Experimental procedures timeline. **(B)** The novel object recognition (NOR) test showing increased discrimination index by thioperamide (THIO). **(C)** The Y maze (YM) test showing increased spontaneous alternation rate by thioperamide. **(D, E)** The Morris water maze (MWM) test showing decreased escape latency on acquisition phase and increased times across the platform on maintenance phase by thioperamide. Data are presented as mean ± SEM. n = 7 for each group. *P <0.05, **P <0.01, ***P < 0.001 in **(B**, **C**, **E)**. **P < 0.01, ***P < 0.001 vs. the sham group; ^#^ *P < * 0.05 vs. the vehicle group in **D**.

Studies show that CCH causes cognitive impairment (Yoshizaki et al., 2008). We investigated the effect of thioperamide on the cognitive impairments in mice, including the novel objection recognition, the Y maze (YM) and the morris water maze (MWM) (**Figure 7A**). The results indicated that in the NOR test, the discrimination index of the vehicle group decreased significantly compared with the sham group (from 12.21% ± 14.38% to −5.10% ± 16.24%, *P* < 0.05, **Figure 7B**), and the thioperamide group significantly increased the discrimination index compared with the vehicle group (from −5.10% ± 16.24% to 26.01% ± 22.26%, *P <* 0.001, **Figure 7B**). In the YM test, the rate of spontaneous alternation decreased significantly in the vehicle group compared with the sham group (from 60.65% ± 11.58% to 40.25% ± 13.89%, *P <* 0.01, **Figure 7C**), and the rate of spontaneous alternation reversed significantly in the thioperamide group compared with the vehicle group (from 40.25% ± 13.89% to 57.62% ± 6.92%, *P <* 0.05, **Figure 7C**). In MWM test, the results indicated that the escape latency increased significantly in the vehicle group on day 4 and day 5 compared with the sham group (*P <*0.01 and *P <* 0.001 respectively, **Figure 7D**), which was significantly reversed by thioperamide on day 5 (*P <* 0.05, **Figure 7D**). Moreover, the times across the platform in the vehicle group decreased significantly compared with the sham group (from 4.89% ± 41.51% to 2.00% ± 61%, *P <* 0.01, **Figure 7E**), which was also significantly reversed by thioperamide (from 2.00% ± 61% to 4.00% ± 41.5%, *P <* 0.05, **Figure 7E**). The results above indicated that thioperamide improved the cognitive impairments induced by CCH.

## Discussion

Deficits in adult neurogenesis may underlie the cognitive deficits in numerous neurological disorders such as depression (Sahay and Hen, 2007), Alzheimer’s disease (Mu and Gage, 2011), Parkinson’s disease (Marxreiter et al., 2013) and stroke (Dillen et al., 2019). Strategies that increase adult neurogenesis have been researched for their therapeutic potential to treat CNS disorders (Zhu et al., 2004; Yau et al., 2014; Hollands et al., 2016; Morello et al., 2018). Inhibition of H3R can protect against traumatic brain injury (TBI)-induced injury by improving neurogenesis (Liao et al., 2019). H3R antagonist increased the adult hippocampal neurogenesis in aged mice (Guilloux et al., 2017). Neurogenesis also plays a significant role in the treatment of CCH (Song et al., 2018). Here, we suggest that thioperamide, a H3R antagonist, promotes proliferation of NE-4C stem cells and enhances neurogenesis in DG and SVZ region in CCH-induced injury. Thioperamide is the most widely used experimental selective H3R antagonist which can promote the production of histamine (Liao et al., 2019). Although thioperamide has high affinity for H4R, it is not expressed abundantly in the CNS. (Witkin and Nelson, 2004). Moreover, it has also been confirmed that the H4R downstream pathways are similar to those described for H3R (Obara et al., 2019). This is the first study suggesting that H3R antagonist may be a novel strategy for treating CCH-induced cognitive deficits targeting neurogenesis.

The previous study suggested that H3R antagonist improves neurogenesis in TBI-induced injury through activating the histaminergic system (Liao et al., 2019). Our findings suggested that thioperamide promoted proliferation of NE-4C stem cells through activating CREB signaling and subsequently promoted the BDNF production. CREB activation in the DG resulting in postnatal hippocampal neurogenesis was first reported in Young et al. (1999). Strategies that could enhance CREB signaling induces increased adult neurogenesis in the SGZ enhanced neurite outgrowth and dendritic branching (Merz et al., 2011). Activating CREB pathway promoted post-ischemic neurogenesis and recovery of neurological functions (Fujioka et al., 2004; Zhu et al., 2004; Zhang et al., 2018). The positive effects of CREB in neurogenesis were inhibited when CREB expression was repressed (Fujioka et al., 2004; Zhang et al., 2016). Consistent with these studies, our research suggested for the first time that H3R inhibitor could regulate proliferation of neural stem cells through activating its downstream signaling protein CREB.

It is widely accepted that BDNF stimulates neurogenesis (Zhang et al., 2011), and increased BDNF levels have been shown to enhance hippocampal neurogenic capacity, resulting in improvements in learning and memory (Hsiao et al., 2014). BDNF was implicated an important role in the pathogenesis of neurological disorders (Nagahara and Tuszynski, 2011; Lu et al., 2013). Environmental enrichment and voluntary exercise enhanced learning and memory by increasing neurogenesis through upregulating BDNF levels (Bekinschtein et al., 2011). BDNF promoted neurogenesis and protected against ischemic injury (Schabitz et al., 2007; Guan et al., 2012). Moreover, CREB phosphorylation promoted the BDNF transcription (Tao et al., 1998; Yan et al., 2016). Therefore, we analyzed the effect of thioperamide on BDNF levels. Our results showed that thioperamide upregulated the BDNF levels, and inhibition of CREB by H89 compromised this effect, suggesting thioperamide promote neural stem cell proliferation through enhancing transcription of BDNF *via* activating CREB. Our results were consistent with study that activation of PKA/CREB signaling showed ameliorated depressive behaviors through upregulating BDNF expression (Xue et al., 2016), showing that strategies activating CREB/BDNF pathway are beneficial to neurogenesis.

Neurotrophins, especially BDNF, are potent modulators of dendritic development. They can act in anterograde, autocrine or paracrine manners to modulate dendritic growth (Baquet et al., 2004; Sanchez et al., 2006). Defect of dendritic morphology have therefore been known to effect the neuronal connectivity (Cline, 2001). In view of the results that thioperamide promoted the BDNF levels, it raises possibility that thioperamide might enhance the dendritic grow. Interestingly, our studies showed that OGD induced a significant decrease in both total dendritic length and dendritic branching, which was reversed by thioperamide. Our results were consistent with research that CREB/BDNF signaling promoted hippocampal CA1 synapse and dendrite formation (Spilker et al., 2016), and the increased BDNF levels was involved in regulating dendritic spine loss (Miguez et al., 2015), implicating an important role of thioperamide on neuronal development by manipulation of BDNF levels.

Mounting evidence suggested that adult neurogenesis plays a crucial role in brain structural plasticity, learning and memory (Franklin and Perrot-Sinal, 2006; Cameron and Glover, 2015; Hollands et al., 2016) and declines with age (Jaroudi et al., 2017), and deficient neurogenesis is accompanied by cognitive dysfunction (Deng et al., 2010; Spinelli et al., 2019). Therefore, endogenous neurogenesis may play an important role in cognition in CNS diseases. Interestingly, our *in vivo* studies suggested for the first time that thioperamide rescued the impaired neurogenesis in both DG and SVZ region on 35 days after CCH. Interestingly, we have observed an increased number of BrdU^+^ cells in both DG and SVZ regions at 2 days after CCH-induced injury, and thioperamide further promoted the cell proliferation. The proliferation of neural stem cells increased on day 2 and decreased on day 35 was consistent with study that ischemic stroke induced enhanced neurogenesis on early stage and deficient neurogenesis on later stage (Jin et al., 2001), suggesting an endogenous protection by increasing neural stem cells against CCH-induced injury on the earlier stage. Moreover, we suggested that H3R antagonist ameliorated the cognitive impairments in CCH-induced injury. Previous studies showed that histidine is protective against CCH-induced injury by promoting neurogenesis in the DG region (Song et al., 2018). These results are consistent with our results that thioperamide enhanced neurogenesis and rescued impaired cognition in CCH-induced injury, suggesting that H3R antagonist might alleviate CCH-induced cognitive dysfunction through enhancing neurogenesis, and of course further studies are still needed to verify the notion.

Our findings revealed the role of H3R antagonists in promoting neurogenesis during CCH, and suggested that H3R antagonism can be a potential therapeutic strategy for CCH. However, further studies remain to be investigated, including the role of BDNF and its related receptors in the effect of thioperamide on neurogenesis and importance of neurogenesis in the effect of thioperamide on cognition in CCH.

## Conclusions

The findings of this study showed that inhibition of H3R promoted proliferation of neural stem cells through CREB/BDNF pathways, and rescued the impairment of cell viability and neuronal morphology of primary neurons against OGD-induced injury. Thioperamide also enhanced neurogenesis in DG and SVZ regions and ameliorated cognitive impairments in CCH-induced injury.

## Data Availability Statement

All datasets generated for this study are included in the article/supplementary material.

## Ethics Statement

This study was carried out in accordance with the recommendations of the Animal Experimentation Committee of Binzhou Medical University and Binzhou Medical University Hospital. The protocol was approved by the Animal Experimentation Committee of Binzhou Medical University and Binzhou Medical University Hospital.

## Author Contributions

HY and SJ designed the experiments. HY, SJ, and NW analyzed the data and wrote the article. HY, SJ, NW, JM, JL, JW, CL, HW, and YL performed the experiments.

## Funding

Work of the authors was supported by grants from the National Natural Science Foundation of China (81500930 and 81402904), the key R&D Plan of Shandong Province (2019GSF108110), the Natural Science Foundation of Shandong Province (ZR2014HQ014 and ZR2014CM038), and the Natural Science Foundation of Shanghai (18ZR1424900).

## Conflict of Interest

The authors declare that the research was conducted in the absence of any commercial or financial relationships that could be construed as a potential conflict of interest. 
